# Reducing delays in time-critical medications for Parkinson’s disease: a multifaceted, multiprofessional quality improvement project

**DOI:** 10.1136/bmjoq-2025-003715

**Published:** 2026-01-30

**Authors:** James Fisher, Charlotte Scott

**Affiliations:** 1 School of Medicine, Newcastle University, Newcastle upon Tyne, UK; 2 Northumbria Healthcare NHS Foundation Trust, North Shields, UK

**Keywords:** Medication safety, Geriatrics, Quality improvement, Healthcare quality improvement, Time-to-Treatment

## Abstract

When people with Parkinson’s disease (PD) are admitted to hospital, control of their symptoms can deteriorate, often due to delayed or incorrect medication administration. The aim of this project was to improve the administration of PD medicines for hospital in-patients in our trust. Specifically, we aimed to administer 95% of PD medicines within 30 minutes of the prescribed time and to eliminate delays of >60 minutes for PD medications.

To achieve these aims, we developed a multifaceted quality improvement project, led by a multidisciplinary team, that ran over a period of 2 years. The outcome measure in this project was the time delay between the time a given PD medicine was scheduled to be administered and the time at which it was recorded as having been administered by nursing staff.

The data were divided into 3 phases: a 6-month baseline phase (March 2022 to September 2022), a 24-month project phase (September 2022 to September 2024) and a 6-month sustain phase (September 2024 to March 2025). Statistical process control (SPC) charts were used to monitor medicine delays over time. Plan-do-study-act methodology was adopted within this project and a variety of interventions were employed throughout the project.

The project demonstrated a significant reduction in delays in medicines administration for patients with PD. The success of our project came from the cultivation of multiprofessional ‘ownership’ of the problem, in combination with an appreciation of the patient’s lived experience, through visualisation of how poor symptom control can impact on a person’s ability to move. Whilst it is not possible to comment on the long-term sustainability of the project, we were encouraged that the changes were maintained throughout the 6-month sustain phase for both medicine administration targets.

**WHAT IS ALREADY KNOWN ON THIS TOPIC**

- It is recognised that delays in the administration of medicines for people with Parkinson’s are common and are associated with worse outcomes. Conversion of oral to non-oral PD medications, for those patients with swallowing problems, is also recognised as a challenging area. We sought to improve the administration of PD medicines for hospital in-patients in our trust through a multifaceted quality improvement project, led by a multidisciplinary team, that ran over a period of 2 years.

**WHAT THIS STUDY ADDS**

- Our project demonstrated a significant reduction in delays in medicines administration. The success of the project came from the cultivation of multiprofessional ‘ownership’ of the problem, in combination with an appreciation of the patient’s lived experience. The availability of an easy-to-use medication calculator helped colleagues address gaps in their knowledge around oral to non-oral medication conversion.

**HOW THIS STUDY MIGHT AFFECT RESEARCH, PRACTICE OR POLICY**

- Many of the interventions we employed were low cost and could conceivably be employed by other centres. For example, Parkinson’s UK’s ‘get it on time’ resources are freely available to healthcare professionals via their website; the medicine conversion calculator is freely available online (pdmedcalc.co.uk).

## Problem

 Globally, Parkinson’s disease (PD) is the second most common neurodegenerative condition, and its prevalence is increasing.^1^ When people with PD are admitted to hospital, it is recognised that control of their symptoms deteriorates, often due to delayed or incorrect medication administration.^2^ Abrupt discontinuation of PD medications can also be extremely dangerous, since it can precipitate a neuroleptic malignant syndrome, which carries high mortality.^3 4^

The aim of this project was to improve the administration of PD medicines for hospital in-patients in our trust. Specifically, we aimed to:

1. Administer 95% of PD medicines within 30 min of the prescribed time.
2. Eliminate delays of >60 min for PD medications.
3. Deliver education and training to hospital staff on the essentials of in-hospital care for patients with PD.

To achieve these aims, we developed a multifaceted quality improvement project, led by a multidisciplinary team, that ran over a period of 2 years. The project was carried out at Northumbria Specialist Emergency Care Hospital (NSECH) in the North East of the United Kingdom. NSECH is part of Northumbria Healthcare NHS Foundation Trust. The trust serves over 500 000 residents across the North East, meaning the local patient population is drawn from a mix of urban and rural settings. The trust employs over 12 000 staff and has had a dedicated Parkinson’s service since the 1990s. In the areas served by our trust, previous studies identified a prevalence rate of 148–178 cases of PD per 100 000.^5 6^ Our project chose to focus on 3 wards—2 acute medical wards and 1 older persons’ medicine ward. These wards were selected as it was recognised that these were the most common wards for patients with PD to be admitted to.

## Background

It is recognised that ensuring on-time administration of PD medicines can be challenging.^2^ The majority of medicines for PD are delivered orally, which can be problematic, given that swallowing problems (dysphagia) are common in PD.^7^ Dysphagia in PD is recognised as being a strong predictor of adverse outcomes, including increased length of stay and higher mortality.^8^ The 2021 National Confidential Enquiry into Patient Outcome and Death (NCEPOD) report examining in-patient dysphagia management for patients with PD therefore makes for alarming reading, since it highlighted widespread sub-optimal care of swallowing.^9^

Sometimes, when PD patients are admitted to hospital, concerns about the safety of their swallowing, or planned procedures, mean they are made ‘nil by mouth’. In this situation, conversion of their existing PD medication to a non-oral form is essential.^10^ Guidance for such medication conversion can be sought from local PD teams and pharmacists, but during overnight or weekend working, this expertise may not be available. It is recognised that making these medication switches can be challenging,^11^ with junior doctors lacking both knowledge and experience of safe conversion of PD medications to non-oral forms.^12^

Previous work has examined how delays to medicine administration in PD could be reduced, with some interventions demonstrating efficacy. These include: educational interventions,^12^,^17^ regular newsletters for staff,^13^ visual prompts for nursing staff,^13^,^1517^ audible prompts for nursing staff,^14^ regular ‘compliance scorecards’ for ward managers,^13^ facilitation of pharmacy medicine reconciliation^15 18 19^ and the development of a specialist unit solely for patients with PD.^16^

Key learning from previous work is that cultivation of multiprofessional ‘ownership’ of the problem, combined with an appreciation of the patient’s lived experience, can be a powerful catalyst for driving change.^14^ Previous work in this area also demonstrates that simple interventions were often more fruitful in driving change.^14^ However, the efficacy of simple interventions can be undermined if ward staff are not fully engaged with their implementation and instead view them as contributing to their workload.^17^ Another barrier to achieving change was the difficulties associated with the conversion of oral to non-oral PD medications.^12^

## Measurement

The outcome measure in this project was the time delay between the time a given PD medicine was scheduled to be administered and the time at which it was recorded as having been administered by nursing staff. These data are recorded within electronic prescribing software—automatic time stamping occurs when medicines are recorded as having been administered. The real-time recording of this data meant that we had confidence that this would provide a valid and reliable measure of medicines delays. Analysis of medicine administration data was undertaken using Triscribe. This software enabled daily extraction of data from the hospital electronic prescribing system and provided this in a consistent database ready for analysis. These data were pooled into monthly periods to allow for comparison over time. Medication data were gathered solely for the following PD medications: amantadine, apomorphine, biperiden, cabergoline, co-beneldopa, co-careldopa, entacapone, opicapone, pergolide, pramipexole, rasagiline, riluzole, ropinirole, rotigotine, selegiline and tolcapone. Process measuring data were also recorded: the number of staff who received the education and training, as well as the number of staff who became ward champions.

The data were divided into 3 phases: a 6-month baseline phase (March 2022 – September 2022), a 24-month project phase (September 2022 to September 2024) and a 6-month sustain phase (September 2024 to March 2025). Statistical process control (SPC) charts were used to monitor medicine delays over time. This served as the primary tool for visualising overall trends. SPC charts, when combined with Nelson rules, are recognised as powerful tools for distinguishing between random variation and meaningful change within quality improvement projects. Control limits were determined, as this enabled normal process variation to be distinguished from significant deviation, thus providing an evaluation of the effectiveness of the project. Annotations to SPC charts marked critical points within the project and helped with visualisation of the timings of interventions in relation to project data.

## Design

During the planning stage of this project, we identified several assumptions about the underlying problem—these informed the subsequent development of the interventions we employed. First, the breadth of symptoms seen in PD means that gold-standard PD care requires multi-disciplinary input. For this reason, the team of professionals involved with this project was drawn from a wide variety of professional backgrounds, enabling their range of expertise to be harnessed. The team consisted of speech and language therapists, nurses, a nurse-led practice development team, pharmacists, pharmacy technicians, patient safety experts, senior doctors and resident doctors. Similarly, the interventions described below were targeted at a multidisciplinary audience, not just to 1 professional group. This was felt to be a key factor in the sustainability of the change—we were striving for ward teams, across all disciplines, to take ownership for this problem at their local level. Patients or the public were not involved in the design, or conduct, or reporting, or dissemination plans of our project.

We also recognised that medicine delays sometimes arose because of delays in the assessment of a patient’s swallow. This initial swallow assessment, when a patient is admitted to a ward, has tended, historically, to be a job undertaken by a member of the nursing team. We hoped to broaden the range of people who were both willing and able to undertake this assessment and to reconceptualise it as not just a ‘nurse job’. We also recognised that the cultivation of this ownership among ward teams required that staff develop a more nuanced understanding of the impact of PD on those living with the disease.^20^ By making patient experience a central component of the teaching intervention, we aimed to build empathy among staff by illustrating to them the challenges faced by individuals with PD when admitted to hospital.

Lastly, we were acutely aware of the immense pressure that ward teams are under. We recognised that delivering extensive teaching for all members of a ward team ‘off-site’ was likely unrealistic, in view of their high workload. Delivery of the teaching in situ was therefore deemed critical to the success of this project. We were also mindful that ward teams may become demoralised by regular messaging that highlights deficiencies in care. This risks undermining motivation to modify practice and subsequent calls to address deficiencies may not result in meaningful change. We therefore sought to avoid any criticism of practice within teaching. Instead, we sought to develop brief, eye-catching teaching that included clear signposting to sources of help when teams were caring for people with PD and to sources of further training.

Interventions were structured and tested through iterative plan-do-study-act cycles:

- A 3-minute duration ‘micro-teaching’ session was delivered by the practice development team to all study wards. This included video footage of a patient with significant walking problems because of a 30 minute delay to their Parkinson’s medication. This was followed by footage of the same person, 30 minutes *after* they had received their medication, which demonstrated profound improvement in his mobility. Footage was displayed to learners on a tablet device, as this enabled the teaching to be delivered anywhere on the ward, thus maximising the number of staff that could be engaged. There were 4 key messages in the micro-teaching: ‘Get it on time’, early assessment of swallow, zero tolerance for missed medicines and inform the PD team that the patient is in hospital. The micro-teaching ran during 2 periods—June 2023 and April 2024, with a total of 169 ward staff receiving the training. As the project ensued, there was recognition that some medicine administration delays on the wards arose due to issues in the emergency department (ED), for example, failure to appreciate swallowing problems. To raise awareness within ED, the micro-teaching was delivered to a further 82 ED staff members during the April 2024 period.
- Resources from Parkinson’s UK were made available to all intervention wards. These included: wipe-clean images of clocks, where a person’s medication timings could be written, that were placed at the bedside to provide a visual prompt to nursing staff; alarm clocks, that ward staff could set to go off when a person’s dose was due; ‘toilet bags’ (small bags used to carry personal hygiene supplies when travelling) given out on discharge, which patients could keep a supply of their PD medicines in, ready in case they were re-admitted to hospital.
- Dysphagia training ran in the trust once a month and was delivered by the speech and language therapy team. Dates of upcoming sessions were shared with staff members who completed the microteaching and were advertised to the multidisciplinary team working on the intervention wards. During the project phase, a total of 30 people from target wards attended this training.
- Updating the internal trust guidelines for management of PD to make them more practicable. The updated version made specific reference to the importance of early assessment of swallowing, getting PD medication on time and the process to be followed if a person were unable to swallow their medicines [November 2022].
- Documentation for bedside swallow assessment, completed electronically (via NerveCentre) was amended. Previously, this assessment had been termed ‘nurse swallow assessment’. To promote completion of this assessment by any qualified member of staff, the word ‘nurse’ was removed [January 2023].
- A clinical safety message was recorded and shared with all staff members within the trust. The safety message covered the 4 key messages of the project [October 2023]
- A grant was secured from Parkinson’s UK, the leading UK PD charity, to update PDMedCalc. PDMedCalc is an online tool, developed by Northumbria Healthcare NHS Foundation Trust, to support staff members when their patients are unable to take their medicines orally^21^ . A patient’s usual oral PD medicine regimen is inputted into the calculator, where it is converted to a levodopa equivalent dose to be delivered via an alternative route—either dispersible co-beneldopa via a naso-gastric (NG) tube or a rotigotine transdermal patch (if an NG is not suitable). This tool was then ratified and approved as a medical device from the Medicines and Healthcare products Regulatory Agency (MHRA) [March 2024].
- Critical medicine wristbands were introduced to 1 acute medicine study ward. This was a bright green wristband given to all patients who had been admitted to hospital and were taking a time-critical medicine, which included medicines for PD [May 2024].
- A screensaver, displaying a summary of the key messages of the project (aligning with the learning outcomes of the micro-teaching), was developed with digital services and was displayed on all trust computers and laptops [June 2024].
- PD ‘ward champions’ were identified on each study ward. These were staff members, from any profession, who had received the micro-teaching and thereafter were identified as having a particular interest in the area. The PD ward champions worked on their own ward where they were tasked with reinforcing the message of the microteaching amongst their ward teams. They were also encouraged to adopt their own local initiatives to meet the goals of our project, with their strategies informed by their more nuanced understanding of the ward and the team with whom they worked. The ongoing involvement of the ward champions was considered critical in ensuring the long-term sustainability of this project [June 2024].

## Strategy

Plan-do-study-act (PDSA) methodology was adopted within this project. PDSA supports continuous improvement in a structured, iterative manner, since its use enables small, incremental changes to be tested and refined over time. The continuous approach to data collection that we adopted enabled ward teams to be kept abreast of the progress they were making—this provided timely feedback and was a catalyst for motivation.

Throughout the duration of the project, the improvement team held regular meetings where progress was reviewed, challenges were considered and upcoming implementation of interventions was agreed. On occasion, these discussions led to a change in direction of the project. For example, as the project ensued, there was a recognition that some medicine delays were occurring due to issues that had arisen before the patient had been transferred from the emergency department (ED) to the ward, for example, failure to recognise swallowing problems. This realisation led to a change in the delivery of the microteaching, such that it was also delivered to staff members in the ED, as described above.

## Results

**Figure 1:**
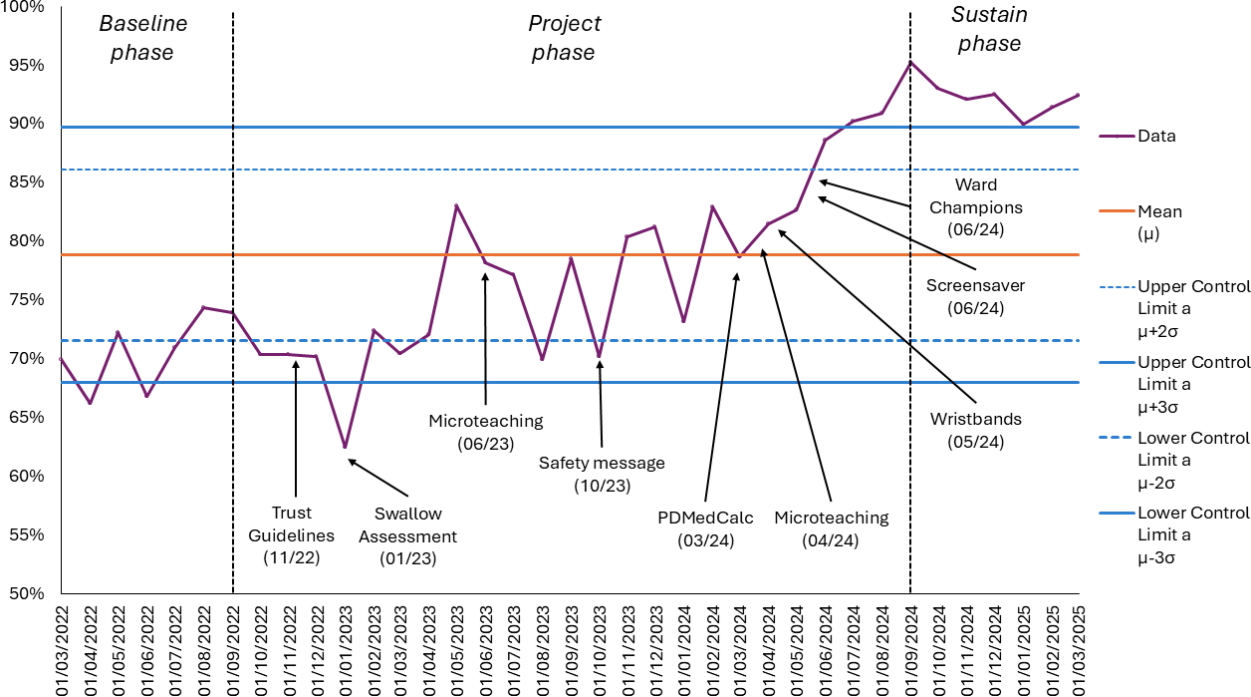
Percentage of Parkinson’s medicines administered within 30 min

**Figure 2:**
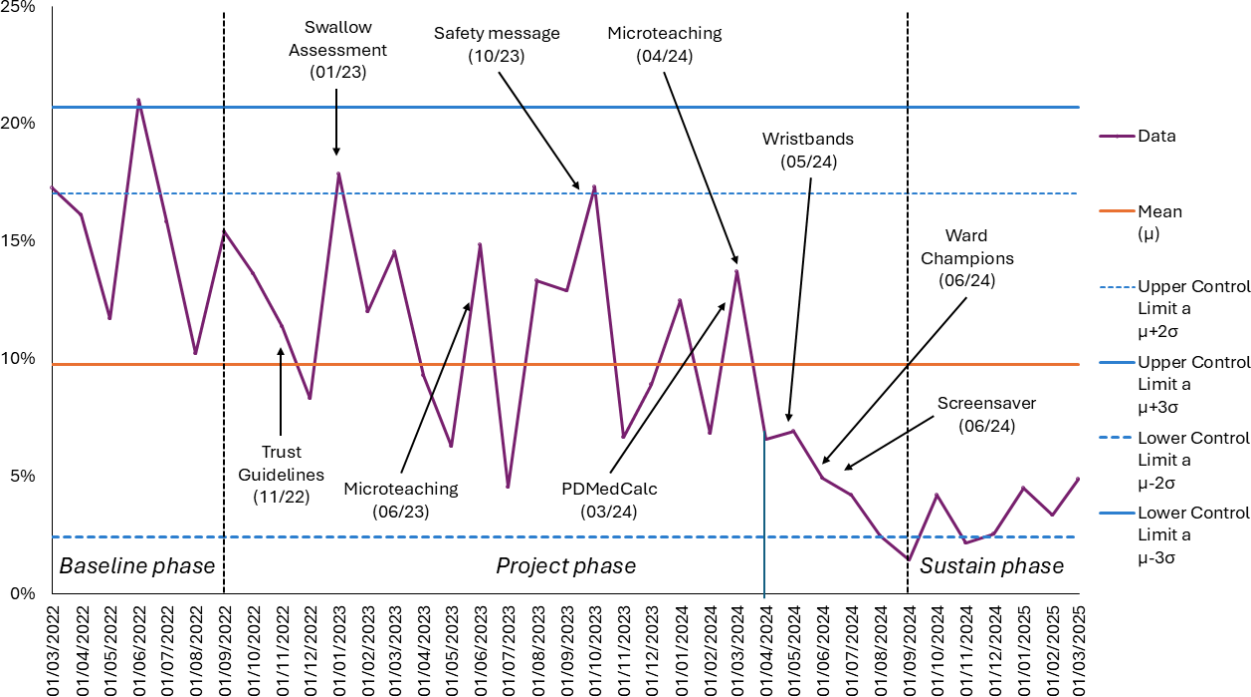
Percentage of Parkinson’s medicines delayed by more than 60 min

Figures1  2 track, for the 3 phases of the project, performance against the 2 project aims that relate to reducing delays in medicines administration. Figure 1 displays the percentage of Parkinson’s medicine administered within 30 min of their prescribed time. Figure 2 displays the percentage of Parkinson’s medicines delayed by more than 60 min. In both charts, control limits are set at ±3 SD relative to the mean.

The data demonstrate a clear improvement in performance from the baseline phase to the project phase, with this improvement persisting into the sustain phase. Visual inspection and application of Nelson rules revealed evidence of special cause variation, suggesting that the process was affected by factors beyond random variation. The microteaching initiative and the development of the ward champions appeared to be the key drivers of the positive change that was observed.

For figure 1, rule 1 (points outside control limits) was triggered in July 2024 (and persisted until March 2025), suggesting unusually high measurement(s). For figure 1, rule 2 (9 or more points in a row on the same side of the mean) was triggered between April 2024 and March 2025, which would indicate sustained change. For figure 1, rule 3 (6 points in a row continuously increasing/decreasing) was triggered between April 2024 and September 2024, demonstrating a steady upwards trend. For figure 2, rule 2 (9 or more points in a row on the same side of the mean) was triggered between April 2024 and March 2025, which would indicate a sustained change.

## Lessons and limitations

A key lesson from this project relates to the educational intervention, the so-called microteaching. We contend that the positive change our data demonstrate supports our initial assertion that brief, in situ training is more likely to engage busy staff in acute settings and is therefore more likely to result in a change in practice. Furthermore, the teaching was distilled into 4 key messages that were reiterated via several routes—the trust safety message, the second round of micro-teaching and the screensaver. We believe that this spaced repetition may have improved recall of our education among busy staff members.

The relatively low attendance at dysphagia training is acknowledged. This may reflect challenges associated with staff obtaining time for training away from their clinical workplace. This finding also supports our assertion that in situ training can be particularly valuable for busy clinical staff.

A limitation of our work is the fact that we restricted our interventions to three acute wards at NSECH. The justification for this was the fact that these were the most common wards for patients with PD to be admitted to. It could be argued that wards which infrequently care for patients with PD may be in greater need of the education and support offered as part of this project. However, it is possible that some of the interventions employed in the project had reach beyond the intervention wards due to their wider visibility within the trust, for example, computer screensaver, trust safety message, time-critical medicine wrist-bands.

A key strength of this project was the engagement of multidisciplinary team members from the point at which the project was conceived. The involvement of professionals with a broad range of backgrounds helped shape the project into something that reached and engaged all members of the ward MDT, as evidenced by the large numbers of staff who received the education. We contend that the inclusive nature of the interventions, when combined with the power of the patient experience seen within the educational videos, helped engender a sense of ownership for the project among ward teams. A further strength of the project related to the method of data collection, which, thanks to its interface with the trust’s electronic prescribing software, enabled rapid and complete data acquisition of medicines administration. A potential confounding factor relates to the process by which medicines administration was recorded. Timings were identified by the electronic time-stamp that was generated when nursing staff recorded that the medicine had been given. We recognise that we cannot be certain, from this time-stamp alone, that this represents the actual time at which the patient took the medicine. Recording this through in-person observation of medication rounds might have enhanced the validity of our data, but such an approach was not feasible because of the scale of our project.

We acknowledge the lack of a control group for direct comparison of data. The use of other non-intervention wards for this purpose was considered, but the small number of PD patients being cared for on such wards rendered data incomparable. Instead, we believe that the 6-month period of data collection on intervention wards (March 2022 to September 2022) provided sufficient baseline data to enable a before–after comparison. We also recognise that the site at which our project was conducted (NSECH) may not be representative of all hospitals. NSECH, opened in 2015, was the first purpose-built emergency care hospital in the UK’s National Health Service (NHS). NSECH is built around centralised emergency care with focus placed on the rapid flow of patients to specialist acute care wards built adjacent to ED in a hub and spoke design. While there are now other similar centres in the UK, this factor may limit generalisability to other in-patient settings. We contend that the interventions in this work are not specific to the model of care employed at NSECH and that they could readily be implemented at other sites and that they may yield similar results. A further limitation of our work is that our outcome measure, timeliness of medication administration, was solely a process measure. We did not consider patient-related outcome measures within our project—exploration of length of stay, the rate of adverse outcomes and patient satisfaction would have enhanced our work. Lastly, we acknowledge that while SPC charts demonstrate improvement associated with the multi-faceted intervention package, the relative contribution of individual components cannot be disentangled due to their continuous and overlapping nature.

## Conclusion

This multifaceted quality improvement project, led by a team with broad multidisciplinary representation, reduced delays in medicines administration for patients with PD. The success of our project came from the cultivation of multiprofessional ‘ownership’ of the problem, in combination with an appreciation of the patient’s lived experience—strategies that have previously been described as powerful potential catalysts for change.^14^ The ability to provide ward teams with near real-time feedback on their progress, as has been described previously,^13^ proved to be a powerful motivating factor. The availability of a simple, easy-to-use medication calculator helped junior doctors address the recognised gap in their knowledge for safe conversion of PD medications to non-oral forms.^12^

The specific aim of this project was to improve the administration of PD medicines for hospital in-patients in our trust. Our first target was to administer 95% of PD medicines within 30 minutes of the prescribed time. While this target was not achieved consistently, there was a significant improvement in performance in this domain, with >90% being achieved throughout the sustain phase. Our second target was to eliminate delays of >60 minutes for PD medications. Reducing this to zero was recognised as a tough challenge. While this numerical target was not achieved, we believe that the data demonstrate that we have made a significant improvement in this domain. Delays of >60 minutes were reduced to <5% and this was maintained throughout the sustain phase. Despite both these numerical targets proving hard to achieve, we opted against adjusting them as we were determined to aspire to the highest standard of care. Our final target, which was completed, was to deliver education and training to hospital staff on the essentials of in-hospital care for patients with PD.

No cost analysis was undertaken as part of this work, so judgements regarding potential cost savings were not possible. Delays to the administration of PD medications do worsen patients’ symptoms, notably their mobility, and this is recognised as prolonging length of hospital stay, which carries high associated costs. Many of the interventions we employed were low cost and could conceivably be employed by other centres. For example, Parkinson’s UK’s ‘get it on time’ resources are freely available to healthcare professionals via their website; the medicine conversion calculator is freely available online (pdmedcalc.co.uk).

While it is not possible to comment on the long-term sustainability of the project, we were encouraged that the changes were maintained throughout the 6-month sustain phase for both medicine administration targets. We continue to nurture the enthusiasm and support the development of the ward champions as we consider them to be ongoing agents of change within ward environments. These staff members may also seed good practice to other settings as and when they move to new working environments. While delivering the microteaching to ED staff, our team realised that similar training for paramedic staff would also be of benefit. This led to the team delivering bespoke education at an North East Ambulance Service away day in September 2024. Work is also ongoing to share the key learning from this project with other centres in our geographical area, through an in-person, multidisciplinary conference, which ran in October 2025. Our aspiration is that this provides a forum for centres to share good practice, and that the key messages from our work may inform quality improvement initiatives at other centres.

## Data Availability

No data are available.
